# Association between Genetic Polymorphisms in Ca_v_2.3 (R-type) Ca^2+^ Channels and Fentanyl Sensitivity in Patients Undergoing Painful Cosmetic Surgery

**DOI:** 10.1371/journal.pone.0070694

**Published:** 2013-08-05

**Authors:** Soichiro Ide, Daisuke Nishizawa, Ken-ichi Fukuda, Shinya Kasai, Junko Hasegawa, Masakazu Hayashida, Masabumi Minami, Kazutaka Ikeda

**Affiliations:** 1 Addictive Substance Project, Tokyo Metropolitan Institute of Medical Science, Tokyo, Japan; 2 Department of Pharmacology, Graduate School of Pharmaceutical Sciences, Hokkaido University, Sapporo, Japan; 3 Department of Oral Health and Clinical Science, Division of Dental Anesthesiology (Orofacial Pain Center/Suidoubashi Hospital), Tokyo Dental College, Tokyo, Japan; 4 Department of Anesthesiology and Pain Medicine, Juntendo University School of Medicine, Tokyo, Japan; Department of Psychiatry, Japan

## Abstract

Individual differences in the sensitivity to fentanyl, a widely used opioid analgesic, lead to different proper doses of fentanyl, which can hamper effective pain treatment. Voltage-activated Ca^2+^ channels (VACCs) play a crucial role in the nervous system by controlling membrane excitability and calcium signaling. Ca_v_2.3 (R-type) VACCs have been especially thought to play critical roles in pain pathways and the analgesic effects of opioids. However, unknown is whether single-nucleotide polymorphisms (SNPs) of the human *CACNA1E* (calcium channel, voltage-dependent, R type, alpha 1E subunit) gene that encodes Ca_v_2.3 VACCs influence the analgesic effects of opioids. Thus, the present study examined associations between fentanyl sensitivity and SNPs in the human *CACNA1E* gene in 355 Japanese patients who underwent painful orofacial cosmetic surgery, including bone dissection. We first conducted linkage disequilibrium (LD) analyses of 223 SNPs in a region that contains the *CACNA1E* gene using genomic samples from 100 patients, and a total of 13 LD blocks with 42 Tag SNPs were observed within and around the *CACNA1E* gene region. In the preliminary study using the same 100 genomic samples, only the rs3845446 A/G SNP was significantly associated with perioperative fentanyl use among these 42 Tag SNPs. In a confirmatory study using the other 255 genomic samples, this SNP was also significantly associated with perioperative fentanyl use. Thus, we further analyzed associations between genotypes of this SNP and all of the clinical data using a total of 355 samples. The rs3845446 A/G SNP was associated with intraoperative fentanyl use, 24 h postoperative fentanyl requirements, and perioperative fentanyl use. Subjects who carried the minor G allele required significantly less fentanyl for pain control compared with subjects who did not carry this allele. Although further validation is needed, the present findings show the possibility of the involvement of *CACNA1E* gene polymorphisms in fentanyl sensitivity.

## Introduction

Voltage-activated Ca^2+^ channels (VACCs) mediate Ca^2+^ entry into cells in response to membrane depolarization and play a crucial role in the nervous system by controlling membrane excitability and calcium signaling [1]. VACCs are composed of a major pore-forming subunit (α1A-I and α1S) and multiple auxiliary subunits (α2-δ, β, and γ). Molecular characterizations have determined that the α1E subunit encodes Ca_v_2.3 (R-type) VACCs [2], [3]. Ca_v_2.3 VACCs are reported to be distributed throughout the central and peripheral nervous systems, including pain pathways [4], [5]. Furthermore, Ca_v_2.3 knockout mice have been reported to show functional deficits in pain perception [6]. Thus, Ca_v_2.3 VACCs may be hypothesized to contribute to pain transmission.

Opioid analgesics, such as fentanyl and morphine, are widely used for the treatment of moderate to severe pain. However, the analgesic efficacy of opioids is well known to vary widely among individuals [7]. Individual differences may be related to various genetic and nongenetic factors, including gender, age, ethnic origin, hepatic or renal function, and mental status [8]. Several studies that used mice that lack the μ-opioid receptor (MOP) [9], [10], [11] have shown that analgesia produced by opioids crucially depends on the level of MOP expression. Furthermore, several single-nucleotide polymorphisms (SNPs) in the *OPRM1* (opioid receptor, mu-1) gene, which encodes the human MOP protein, have been reported to lead to differences in the analgesic efficacy of opioids [12].

Voltage-activated Ca^2+^ channels have also been considered to play important roles in the analgesic effects of and tolerance to opioids. Ca^2+^ influx modulators have been shown to affect both the antinociceptive effects of and tolerance to morphine [13], [14], [15]. Moreover, Ca_v_2.3 knockout mice show enhanced analgesic effects of morphine [16]. One of the supraspinal analgesic mechanisms by which opioids are known to disinhibit the endogenous descending antinociceptive pathway is via inhibition of γ-aminobutyric acid (GABA) neurons in the periaqueductal grey (PAG). The relatively high expression level of Ca_v_2.3 in the PAG has also been reported [6]. Although the precise mechanism is still unknown, Ca_v_2.3 in the PAG could affect the activity of the endogenous descending antinociceptive pathway by regulating the release of GABA or other endogenous neurotransmitters. Thus, the expression level of or functional changes in Ca_v_2.3 may cause differences in the analgesic efficacy of opioids.

Human Ca_v_2.3 is encoded by the *CACNA1E* (calcium channel, voltage-dependent, R type, alpha 1E subunit) gene, which is located on chromosome 1q25-31. Many SNPs have been identified in the *CACNA1E* gene, and some of these SNPs have been reported to be associated with type 2 diabetes [17], [18]. Unknown is whether genetic polymorphisms in the *CACNA1E* gene have any association with pain sensitivity or opioid analgesia. In contrast to animal studies that use standardized pain tests, the analgesic effects of opioids in humans are usually evaluated in patients with actual pain, particularly cancer pain or acute postoperative pain [12]. Patients with acute postoperative pain following standardized surgical procedures may be more optimal subjects for investigating gene-opioid effect relationships [7], [19]. Therefore, the present study examined whether SNPs in the *CACNA1E* gene affect pain sensitivity and the analgesic effects of fentanyl, one of the most commonly used opioid analgesics, evaluated by a standardized pain test and fentanyl requirements in healthy Japanese subjects who underwent uniform surgical procedures.

## Materials and Methods

### Ethics Statement

The study protocol was approved by the Institutional Review Board, Tokyo Dental College, Chiba, Japan, and the Institutional Review Board, Tokyo Metropolitan Institute of Medical Science, Tokyo, Japan. Written informed consent was obtained from all of the patients and from parents if required.

### Patients

Enrolled in the study were 355 healthy patients (American Society of Anesthesiologists Physical Status I, age 15–52 years, 125 males and 230 females) who were scheduled to undergo cosmetic orthognathic surgery (mandibular sagittal split ramus osteotomy) for mandibular prognathism at Tokyo Dental College Suidoubashi Hospital. Patients with chronic pain, those taking pain medication, and those who had experienced Raynaud’s phenomenon were excluded.

### Preoperative Cold Pressor-induced Pain Test

Patients were premedicated with oral diazepam, 5 mg, and oral famotidine, 150 mg, 90 min before the induction of anesthesia. Patients had an intravenous (i.v.) line on the forearm on their nondominant side. The temperature in the operating room was maintained at 26°C. The cold pressor-induced pain test was then performed before and 3 min after an i.v. bolus injection of fentanyl, 2 µg/kg, as previously described [19], [20]. Briefly, crushed ice cubes and cold water were blended 15 min before the test in a 1 L isolated tank, and the mixture was stirred immediately before each test to ensure uniform temperature distribution (0°C) within the tank. The dominant hand was immersed up to the wrist. Patients were instructed to keep the hand calm in the ice-cold water and withdraw it as soon as they perceived any pain. All of the patients were administered the test by the same investigator. The baseline latency to pain perception, defined as the time of immersion of the hand in the ice water, before an i.v. injection of fentanyl (PPLpre) was recorded. A cut-off point of 150 s was set to avoid tissue damage. The hand was warmed with a hair dryer as soon as it was withdrawn from the ice water until the sensation of cold was completely abolished. Patients then received i.v. fentanyl, 2 µg/kg. Three minutes after the injection, the pain perception latency of the dominant hand (PPLpost) was measured again. The analgesic effect of fentanyl in the preoperative cold pressor-induced pain test was evaluated simply as the difference between PPLpost and PPLpre (PPLpost - PPLpre).

### Anesthesia and Surgery

After the cold pressor-induced pain test ended, general anesthesia was induced with a target-controlled infusion (TCI) of propofol using a TCI pump (TE-371, Terumo, Tokyo, Japan). Vecuronium, 0.1 mg/kg, was administered to facilitate nasotracheal intubation. After the induction of anesthesia, 10 ml of venous blood was sampled for the preparation of DNA specimens. General anesthesia was maintained with propofol at a target blood concentration of 4–6 µg/ml. Vecuronium was administered at a rate of 0.08 mg/kg/h. The lungs were ventilated with oxygen-enriched air. Local anesthesia was performed on the right side of the surgical field with 8 ml of 2% lidocaine that contained epinephrine, 12.5 µg/ml, and right mandibular ramus osteotomy was performed. Local anesthesia was then performed on the left side, and left mandibular ramus osteotomy was performed. The bilateral mandibular bone segments were fixed in appropriate positions. Whenever systolic blood pressure or heart rate exceeded +20% of the preinduction value during surgery, i.v. fentanyl, 1 µg/kg, was administered.

### Postoperative Pain Management

At the end of the surgery, rectal diclofenac sodium, 50 mg, and i.v. dexamethasone, 8 mg, were administered at the request of surgeons to prevent postoperative orofacial edema/swelling. After emergence from anesthesia and tracheal extubation, droperidol, 1.25 mg, was administered i.v. to prevent nausea/vomiting, and i.v. patient-controlled analgesia (PCA) with a fentanyl-droperidol combination (2 mg fentanyl and 5 mg droperidol diluted in normal saline in a total volume of 50 ml) commenced using a CADD-Legacy PCA pump (Smiths Medical Japan, Tokyo, Japan). A bolus dose of fentanyl, 20 µg, on demand and a lockout time of 10 min were set. Continuous background infusion was not employed. Droperidol was coadministered with fentanyl to prevent nausea/vomiting because our preliminary study showed a high incidence (up to 30%) of nausea/vomiting with PCA fentanyl in young females. Patient-controlled analgesia continued for 24 h postoperatively. In the case of treatment-refractory adverse effects or inadequate analgesia, PCA was discontinued, and rectal diclofenac sodium, 50 mg, was prescribed as a rescue analgesic as required (two patients required a rescue analgesic only once). The intensity of spontaneous pain was assessed 3 and 24 h postoperatively using a 100-mm visual analog scale (VAS), with 0 mm indicating no pain and 100 mm indicating the worst pain imaginable. Intraoperative fentanyl use and postoperative PCA fentanyl use during the first 24 h postoperative period were recorded. Doses of fentanyl administered intraoperatively and postoperatively were normalized to body weight. Additionally, perioperative fentanyl use was calculated as the sum of intraoperative fentanyl use and postoperative fentanyl use because the analgesic effect of the intermediate-acting opioid fentanyl, administered pre- and intraoperatively, could outlast the duration of surgery and thus affect postoperative fentanyl use, especially in patients who received large doses of fentanyl intraoperatively. Therefore, in the present study, we considered perioperative fentanyl use an appropriate indicator of fentanyl analgesia in addition to postoperative fentanyl use.

### Genotyping Procedures and Linkage Disequilibrium Analysis

Genomic DNA was extracted from whole-blood samples using standard procedures. The extracted DNA was dissolved in TE buffer (10 mM tris-HCl, 1 mM ethylenediaminetetraacetic acid, pH 8.0). The DNA concentration was adjusted to 5–50 ng/µl for genotyping individual SNPs or 100 ng/µl for whole-genome genotyping using a NanoDrop ND-1000 Spectrophotometer (NanoDrop Technologies, Wilmington, DE).

For the analysis of SNPs within and around the *CACNA1E* gene region, genotype data from whole-genome genotyping were used. Briefly, whole-genome genotyping was performed using Infinium assay II and an iScan system (Illumina, San Diego, CA) according to the manufacturer’s instructions. Five kinds of BeadChips were used to genotype 40, 67, 6, 119, and 123 samples, respectively: HumanHap300 (total markers: 317,503), HumanHap300-Duo (total markers: 318,237), Human610-Quad v1 (total markers: 620,901), Human1M v1.0 (total markers: 1,072,820), and Human 1M-Duo v3 (total markers: 1,199,187). Some BeadChips included a number of probes specific to copy number variation markers, but most were for SNP markers on the human autosome or sex chromosome. Approximately 300,000 SNP markers were commonly included in all of the BeadChips. After the whole-genome genotyping, the data for genotyped samples were analyzed using BeadStudio or GenomeStudio with the Genotyping module v3.3.7 (Illumina) to evaluate the quality of the results, and the genotype data for all of the SNPs with *CACNA1E* gene annotation were extracted. In the data-cleaning process, markers that had “Cluster sep” values (i.e., an index of genotype cluster separation) less than 0.4 and three genotype clusters that were not separate from one another were excluded from the subsequent association study.

Single-nucleotide polymorphisms for the association studies were selected based on recently advanced tagging strategies [21], [22], [23]. To identify relationships between the SNPs used in the study, linkage disequilibrium (LD) analysis was performed in 223 SNPs that were in the approximately 640 kbp region that contained the *CACNA1E* gene, among 1,199,187 markers of the Human 1M-Duo v3 Bead Chip for 100 samples using Haploview v.4.2 [24]. For the estimation of LD strength between the SNPs, the commonly used *D′* and *r^2^* values were pairwise calculated using the genotype dataset of each SNP. Linkage disequilibrium blocks were defined among the SNPs that showed “strong LD,” based on the default algorithm of Gabriel et al. [25], in which the upper and lower 95% confidence limits on *D′* for strong LD were set at 0.98 and 0.7, respectively. Tag SNPs in the LD block were consequently determined using Tagger software with default settings, which is incorporated in Haploview and has been detailed in a previous report [23].

### Statistical Analysis

Parametric and nonparametric data are expressed as mean ± SD and median [interquartile range], respectively. The statistical analysis was performed using IBM SPSS v.20.0.0 software (IBM, Tokyo, Japan). In the present study, none of the clinically measured endpoints that were related to pain sensitivity (i.e., PPLpre) or fentanyl analgesia (i.e., analgesia measured with the preoperative cold pressor test, perioperative fentanyl use, and VAS scores at 3 and 24 h postoperatively) were normally distributed. Therefore, nonparametric analyses, including the Mann-Whitney *U*-test, Kruskal-Wallis test (with Steel-Dwass multiple comparison tests), or Spearman’s rank correlation test, were used to detect possible associations between any of the clinical or genomic parameters (e.g., sex, age, and genotypes of the Tag SNP) and clinical endpoints related to pain sensitivity or the analgesic effects of fentanyl. Many factors other than the genotypes of the screened SNPs (e.g., age and sex) may also influence the analgesic effects of fentanyl. Therefore, when a significant association between a genotype and clinical endpoint was found, factors other than genotype were compared between genotypes using nonparametric analyses according to the types of data to evaluate whether the genotype groups were controlled by other factors that might affect pain sensitivity, the analgesic effects of fentanyl, or fentanyl requirements, including age and sex. Furthermore, we conducted additional multivariate covariate analyses using the Stepwise method (independent variables: SNP genotypes, sex, and age), although the nonparametric distributions of most of our data were not suitable for the application of such parametric techniques. Values of *p*<0.05 were considered statistically significant. The sample size of the present nonparametric data was higher than the estimated size that possesses statistical power (1 minus type II error probability) of 99% for the Cohen’s conventional “medium” effect size of 0.25, when power analysis was performed for analysis of variance with three genotype groups using G*Power v.3.1.3 [26].

## Results

**Figure 1:**
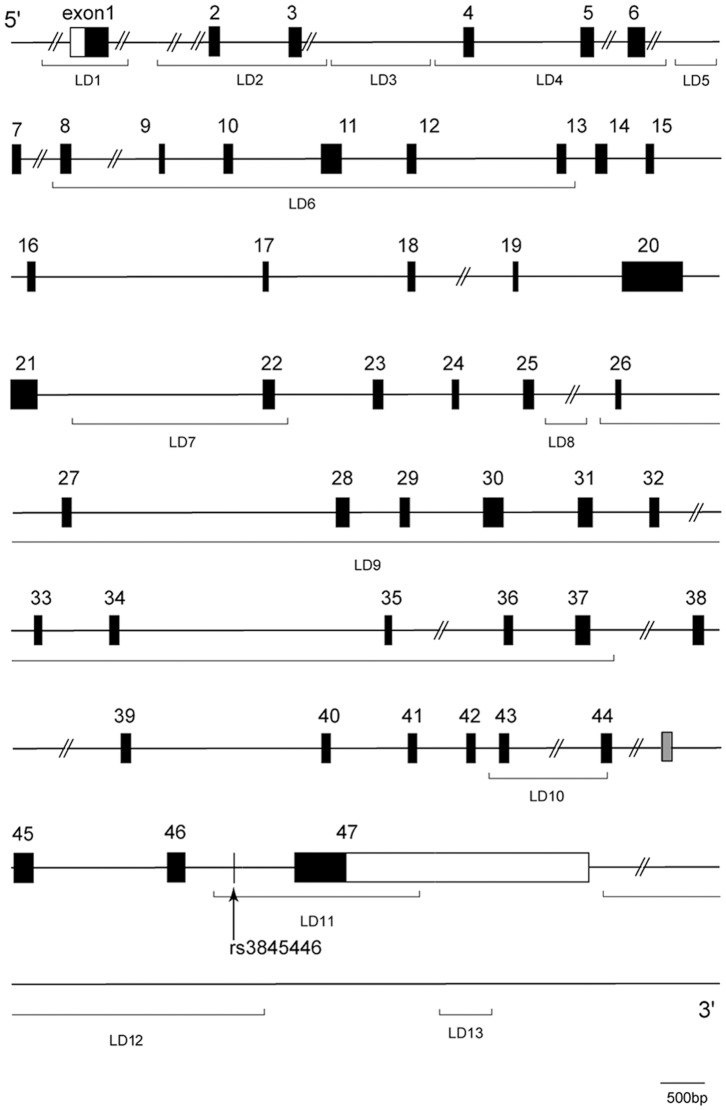
Genomic scheme of the *CACNA1E* gene. Boxes represent exons (black boxes indicate coding regions of major expression type of Ca_v_2.3 [Ca_v_2.3c (α1E-3), NM_000721.3]; gray box indicates exon in the reported major splicing variant isoform [Ca_v_2.3e (α1Ee), NM_001205293.1]; white boxes indicate untranslated regions) [33].

First, to identify the LD blocks in the approximately 640 kbp region that contains the *CACNA1E* gene, 223 SNPs among 1,199,187 markers included in the whole-genome genotyping (Human 1M-Duo v3 Bead Chip) were tested using genomic samples from 100 Japanese patients (Fig. S1). A total of 13 LD blocks, LD1-13, were observed within and around the *CACNA1E* gene region (the exon, intron, and approximately 3 kbp 5′-flanking region and 50 kbp 3′-flanking region of the *CACNA1E* gene [approximately 380 kbp]; Fig. 1), and 42 Tag SNPs were selected in this region (Table 1). In the preliminary study, we analyzed associations between the genotypes of 42 Tag SNPs and perioperative fentanyl use using the same 100 samples. Among these SNPs, only one SNP (rs3845446) in LD11 had a significant association with perioperative fentanyl use (*p* = 0.032, Kruskal-Wallis test; Table 1 and 2). Furthermore, the rs3845446 SNP also had a significant association with perioperative fentanyl use (*p* = 0.033, Kruskal-Wallis test; Table 2) in the confirmatory study using the other 255 samples. Therefore, we further analyzed the associations between the genotypes of the rs3845446 SNP and all of the clinical data using a total of 355 samples. The genotype distributions of the rs3845446 SNP in the patients were AA (168 [47.3%]), AG (148 [41.7%]), and GG (39 [11.0%]). This genotype frequency was in Hardy-Weinberg equilibrium.

**Table 1:** Allelic frequencies and associations with perioperative fentanyl use of SNPs in 13 LD blocks in the *CACNA1E* gene.

LDnumber	SNP name	Major:Minorallele	Allelicfrequency	*p-* value	LDnumber	SNP name	Major:Minorallele	Allelicfrequency	*p-* value
1	rs633143*	G:A	0.100	0.433	6	rs3766988	A:G	0.015	–
	rs2877651*	G:A	0.090	0.491		rs7513540	G:T	0.205	–
	rs556321	A:G	0.187	–		rs3845444	T:G	0.205	–
	rs553042*	T:C	0.365	0.646		rs4306091	A:G	0.015	–
	rs12060765	G:T	0.090	–		rs7540850	C:T	0.235	–
	rs943795	T:C	0.365	–		rs2253388	C:T	0.235	–
2	rs576006	A:G	0.309	–		rs4651112	G:A	0.195	–
	rs12405860*	A:G	0.190	0.662	7	rs12139677*	T:G	0.370	0.402
	rs558994*	G:A	0.295	0.882		rs3845445	T:C	0.045	–
	rs17494681*	C:T	0.110	0.087		rs2280866*	G:A	0.305	0.522
	rs681271*	C:T	0.485	0.927	8	rs3767002*	C:T	0.180	0.399
	rs4126690*	C:A	0.165	0.435		rs16858051	T:C	0.015	–
	rs517209*	C:A	0.460	0.468		rs3753748*	G:A	0.195	0.609
3	rs589082	C:A	0.485	–		rs3767003*	A:G	0.240	0.957
	rs10910948*	T:C	0.120	0.218	9	rs3767004*	G:A	0.275	0.867
	rs16857509	A:G	0.010	–		rs704332*	T:C	0.240	0.957
	rs625226*	A:G	0.125	0.440		rs704331*	A:G	0.390	0.626
	rs4146634*	A:G	0.485	0.783		rs4652678*	T:C	0.060	0.112
	rs2225875*	C:T	0.205	0.103		rs704329*	G:A	0.455	0.990
	rs6681017	C:T	0.120	–		rs4652679	G:A	0.060	–
	rs1933049	G:A	0.480	–		rs697260	G:A	0.175	–
	rs12024842	A:G	0.270	–		rs199922	A:G	0.045	–
	rs4652663	T:C	0.120	–		rs199923	G:A	0.060	–
	rs11580052	G:A	0.485	–		rs199930	C:T	0.060	–
	rs7524309	A:G	0.215	–		rs2280868*	C:A	0.185	0.796
	rs10797724	G:A	0.270	–	10	rs473200*	C:T	0.265	0.638
4	rs10797729*	G:A	0.265	0.287		rs601059	C:T	0.270	–
	rs10797730*	C:T	0.295	0.077		rs704326*	C:T	0.450	0.296
	rs1999838	A:G	0.295	–	11	rs3845446*	A:G	0.335	0.032
	rs7511748	T:G	0.270	–		rs3753752	C:T	0.335	–
	rs12135959	G:A	0.300	–		rs2280869	T:C	0.335	–
	rs3856090	C:T	0.295	–	12	rs12045458*	A:G	0.105	0.727
	rs10494540	G:T	0.300	–		rs7513685*	G:A	0.095	0.177
	rs10494541	G:A	0.005	–		rs610100*	C:A	0.490	0.341
	rs12138634	G:T	0.300	–		rs480752*	C:A	0.055	0.192
	rs12239392	G:A	0.030	–		rs12130868*	C:T	0.400	0.422
	rs2877652	T:C	0.030	–		rs1281194	A:G	0.480	–
	rs6684423	T:G	0.031	–		rs12136390	C:T	0.395	–
	rs3856094*	G:A	0.250	0.246		rs585315*	C:T	0.395	0.601
5	rs199943*	A:C	0.050	0.244		rs12071191	G:A	0.285	–
	rs12071300	T:G	0.050	–		rs486003	G:A	0.395	–
6	rs199916*	G:A	0.190	0.794		rs695072	T:C	0.085	–
	rs3820260	C:T	0.040	–		rs13375273	G:A	0.490	–
	rs3766980*	C:T	0.245	0.886		rs4465155	C:T	0.425	–
	rs3753737*	A:G	0.205	0.969		rs7533297	T:C	0.405	–
	rs10910979	C:T	0.245	–		rs7535666	T:G	0.110	–
	rs3845441	T:C	0.190	–	13	rs598714*	A:C	0.480	0.983
	rs4652673	A:G	0.205	–		rs677618*	T:C	0.375	0.581

* Tag SNPs in the LD blocks (selected using Tagger software with default settings; *r^2^*>0.8). *p*-value, association with perioperative fentanyl use (Kruskal-Wallis test).

**Table 2:** Association between the rs3845446 A/G SNP and perioperative fentanyl use in the preliminary and confirmatory study.

	Genotypes	Numbers	Perioperative fentanyl use (Median)	*p*-value
Preliminary study	AA	46	6.80	0.032
	AG	41	6.83	
	GG	13	5.06	
Confirmatory study	AA	122	8.00	0.033
	AG	107	7.05	
	GG	26	6.61	

*p*-values were calculated using Kruskal-Wallis test.

Of the 355 Japanese patients who enrolled in the study, 353 completed the study. The data from the preoperative cold pressor-induced pain test or postoperative pain management could not be obtained for two patients. The patients’ clinical data are summarized in Table 3. In the preoperative cold pressor-induced pain test, fentanyl (2 µg/kg) increased pain perception latency (PPLpost *vs.* PPLpre, *p*<0.0001, paired *t*-test; Table 3).

**Table 3:** Patient demographic and clinical data.

	All patients	Male	Female
Number of subjects	355	125	230
Age (years)	25.9±7.6 (15–52)	24.5±6.9	26.6±7.9
Body weight (kg)	57.6±10.9 (38–128)	65.9±11.2	53.1±7.5
PPLpre (s)	14 [9], [23] (2–150)	15 [10], [26]	14 [9], [22]
PPLpost (s)	28 [16, 53] (4–150)	35 [19, 70]	26 [15, 48]*
Analgesic effect (PPLpost-PPLpre) (s)	12 [4, 35] (−21 – +143)	15 [5, 40]	10 [4], [29] ( * ^)^
Intraoperative fentanyl use (µg/kg)	4.4 [3.2, 5.9] (0–13.6)	4.0 [2.9, 5.3]	4.6 [3.4, 6.1]*
24 h postoperative fentanyl use (µg/kg)	2.3 [1.0, 4.2] (0–13.8)	1.8 [0.6, 4.0]	2.4 [1.4, 4.3]( * ^)^
Perioperative fentanyl use (µg/kg)	6.9 [5.2, 9.1] (0.8–24.2)	6.4 [4.7, 8.5]	7.2 [5.8, 9.3]*
VAS pain score at 3 h (mm)	27 [15, 50] (0–90)	26 [15, 50]	28 [15, 50]
VAS pain score at 24 h (mm)	25 [10, 42] (0–85)	25 [10, 40]	25 [10, 42]

The data are expressed as numbers, mean ± SD (range), or median [interquartile range].

( *^)^ *p*<0.1,

* *p*<0.05, compared with male subjects.

**Figure 2:**
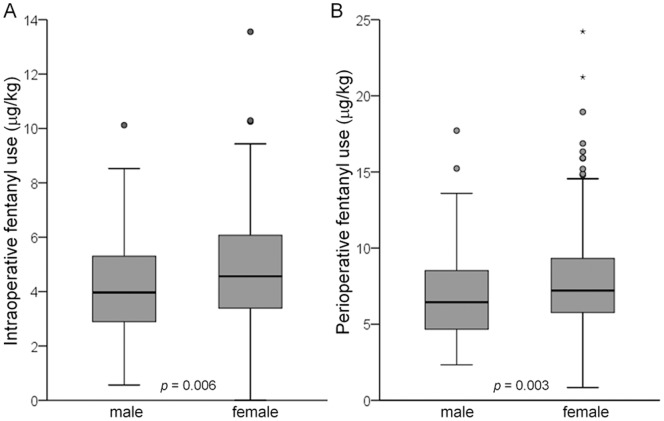
Associations between sex and fentanyl use. Associations between sex (male, *n* = 125; female, *n* = 230) and (A) intraoperative fentanyl use and (B) perioperative fentanyl use. The data are expressed by box and whisker plots. The upper and lower ends of the boxes represent the 75^th^ and 25^th^ percentiles, respectively. Whiskers represent the highest and lowest values that are not outliers or extreme values. Outliers (i.e., values that are between 1.5- and 3-times the interquartile range) and extreme values (i.e., values that are more than 3-times the interquartile range) are represented by circles and starbursts beyond the whiskers, respectively. The median is depicted by a solid line in the box.

**Figure 3:**
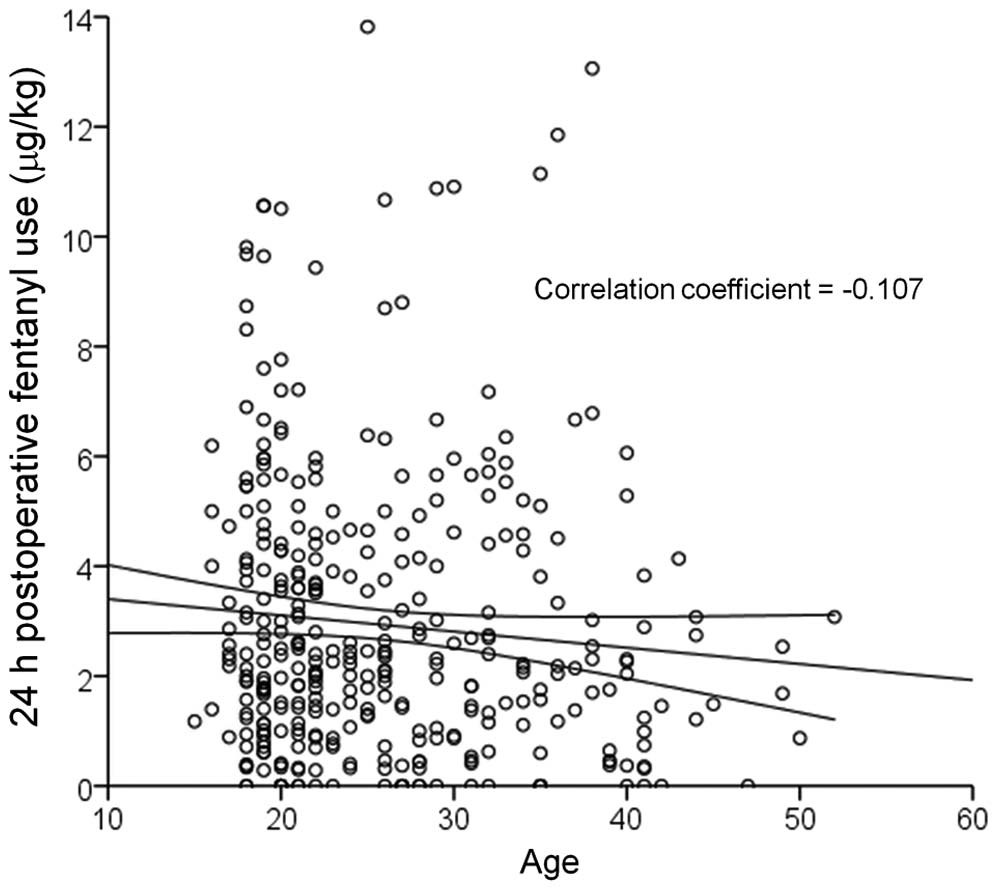
Association between age and fentanyl use. The scatterplot shows the association between age and 24 h postoperative fentanyl use. Each point represents an individual patient.

The Mann-Whitney *U*-test revealed that although sex had no significant association with PPLpre or VAS at 3 or 24 h, the analgesic effect of fentanyl in the cold pressor-induced pain test (PPLpost - PPLpre) tended to be greater in males than in females (*p* = 0.062; Table 3), and 24 h postoperative fentanyl use tended to be less in males than in females (*p* = 0.052; Table 3). Furthermore, intraoperative fentanyl use and perioperative fentanyl use were significantly less in males than in females (*p* = 0.006 and 0.003, respectively; Table 3, Fig. 2). Spearman’s rank correlation test revealed that age had a significant association with 24 h postoperative fentanyl use (*p* = 0.044; Fig. 3) but not with any other clinical endpoints (data not shown). Older age was associated with less fentanyl use for postoperative pain management.

**Figure 4:**
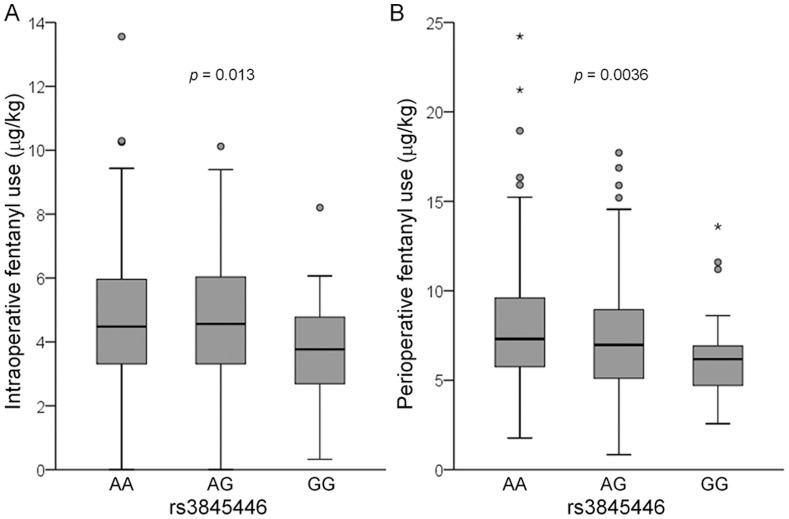
Associations between genotypes of the rs3845446 A/G SNP and fentanyl use. Associations between genotypes of the rs3845446 A/G SNP (AA, *n* = 168; AG, *n* = 148; GG, *n* = 39) and (A) intraoperative fentanyl use and (B) perioperative fentanyl use. The data are expressed by box and whisker plots. The upper and lower ends of the boxes represent the 75^th^ and 25^th^ percentiles, respectively. Whiskers represent the highest and lowest values that are not outliers or extreme values. Outliers and extreme values are represented by circles and starbursts beyond the whiskers, respectively. The median is depicted by a solid line in the box.

The Kruskal-Wallis test revealed that intraoperative fentanyl use (*p* = 0.013), 24 h postoperative fentanyl use (*p* = 0.042), and perioperative fentanyl use (*p* = 0.0036) were significantly associated with genotypes of the rs3845446 SNP (Fig. 4). Steel-Dwass multiple comparisons tests also revealed that subjects who carried the minor G allele of the rs3845446 A/G SNP needed significantly less intraoperative fentanyl and perioperative fentanyl (*p*<0.05) for pain management than those who did not carry this allele (Table 4). The rs3845446 A/G SNP had no significant association with PPLpre, the analgesic effect of fentanyl, or VAS scores at 3 or 24 h (Table 4). The Kruskal-Wallis test revealed no significant differences in age or sex among the subjects who carried different genotypes in the rs3845446 A/G SNP (Table 4). Thus, the differences in fentanyl use among the genotype groups could not be controlled by age and sex. Furthermore, multivariate regression analyses showed that the rs3845446 A/G SNP was retained as an independent predictor of intraoperative fentanyl use (*F*_2,351_ = 6.36, *p* = 0.002), 24 h postoperative fentanyl use (*F*_1,352_ = 6.25, *p* = 0.013), and perioperative fentanyl use (*F*_2,351_ = 9.37, *p*<0.001; significant variables are shown in Table 5).

**Table 4:** Clinical data of genotypes of the rs3845446 SNP.

SNP name	rs3845446		
*Genotype*	*AA*	*AG*	*GG*
Number of subjects(male/female)	55/113	57/91	13/26
Age (years)	25.5±7.3	26.2±7.9	26.7±8.1
PPLpre (s)	14 [9], [23]	15 [10], [24]	14 [8], [21]
PPLpost (s)	28 [16, 58]	26 [16, 53]	27 [16, 43]
Analgesic effect (PPLpost-PPLpre) (s)	13 [5, 38]	11 [4, 35]	11 [4], [28]
Intraoperative fentanyluse (µg/kg)	4.5 [3.3, 6.0]	4.6 [3.3, 6.0]	3.8 [2.7, 4.8]*
24 h postoperativefentanyl use (µg/kg)	2.6 [1.2, 4.6]	2.0 [1.1, 3.6]	2.1 [0.5, 3.9]
Perioperative fentanyluse (µg/kg)	7.3 [5.8, 9.6]	7.0 [5.1, 9.0]	6.2 [4.7, 6.9]*
VAS pain score at 3 h (mm)	26 [15, 50]	30 [18, 50]	24 [10, 43]
VAS pain score at 24 h (mm)	25 [12, 40]	25 [9, 48]	21 [10, 38]

The data are expressed as numbers, mean ± SD (range), or median [interquartile range].

* *p*<0.05, compared with subjects who did not carry the minor allele (Steel-Dwass multiple comparisons tests).

**Table 5:** Multivariate regression models of factors that predict interindividual differences in fentanyl sensitivity.

Predictive variable	Beta	*p*-value
Intraoperative analgesic use (adjusted *r^2^* = 0.029)		
Genotypes of rs3845446 SNP	−0.113	0.031
Sex	0.146	0.006
24 h postoperative fentanyl use (adjusted *r^2^* = 0.015)		
Genotypes of rs3845446 SNP	−0.132	0.013
Perioperative fentanyl use (adjusted *r^2^* = 0.045)		
Genotypes of rs3845446 SNP	−0.173	0.001
Sex	0.139	0.008

Multivariate regression analysis was performed using the Stepwise method (independent variables: Genotypes of rs3845446 SNP, sex, and age). Beta represents the regression coefficient.

## Discussion

We studied patients who underwent mandibular sagittal split ramus osteotomy. Subjects who undergo this cosmetic surgery are usually young and healthy. The operation causes considerable perioperative pain that arises from the dissected mandibular bone, and the surgical technique is highly standardized at our institute. We conducted a standardized pain test before the induction of general anesthesia in opioid-naive subjects without pain. Using these ideal subjects and methods, we found that intraoperative fentanyl use, 24 h postoperative fentanyl use, and perioperative fentanyl use decreased in subjects who carried the minor G allele of the rs3845446 A/G SNP compared with subjects who did not carry this allele in the *CACNA1E* gene. These results suggest that the rs3845446 A/G SNP or other polymorphisms in the same LD11 region could affect the analgesic effects of opioids. Although we analyzed five types of BeadChips merged together in the present study because the sample sizes were small for all the five datasets and they all presumably had Japanese ancestry, performing a meta-analysis is usually better than merging. Moreover, the sample size of the present association study was also quite small. Thus, further studies that have a greater number of samples might be required to reveal the influences of these polymorphisms in the *CACNA1E* gene. PPLpre and the analgesic effects of fentanyl evaluated with the cold pressor test were not associated with the genotypes of the rs3845446 A/G SNP. Although further validation is needed, cold pain sensitivity and the analgesic effects of opioids for acute cold pain might not be associated with genetic polymorphisms in the *CACNA1E* gene. Furthermore, no significant association was found between the genotypes of the rs3845446 A/G SNP and VAS scores, indicating that comparable levels of postoperative analgesia were achieved with PCA fentanyl in our patients, regardless of genotype. The analgesic effects of fentanyl tended to decrease, and fentanyl use for pain management increased in females compared with males, consistent with our previous findings [19]. We also found that older age was significantly associated with less 24 h postoperative fentanyl use. The present results of the influence of demographic factors on pain sensitivity and opioid analgesia were nearly consistent with a recent well-controlled twin study [27]. However, because no significant differences in age or sex were found among the subjects who carried the different genotypes in the rs3845446 A/G SNP, the differences in fentanyl use among the genotype groups in the rs3845446 A/G SNP could not be controlled by these covariates. Furthermore, multivariate regression analyses also showed that the rs3845446 A/G SNP was retained as an independent predictor of fentanyl requirements for pain management.

The therapeutic potential of VACCs in pain management has been the subject of intensive recent investigations [28], [29]. Ca_v_2.3 (R-type) VACCs are classified as “resistant” to blockers of L-, N-, P-, and Q-type Ca^2+^ channels and have been poorly investigated compared with these types of channels. The development of mice that lack Ca_v_2.3 VACCs [6] and the selective Ca_v_2.3 VACC antagonist SNX-482 derived from tarantula venom [30] have made possible the discovery of the direct contribution of Ca_v_2.3 VACCs to pain transmission and opioid analgesia. Ca_v_2.3 VACC knockout mice showed normal pain sensitivity to acute mechanical, thermal, and chemical stimuli but exhibited reduced responsiveness to somatic inflammatory stimulation [6]. Blockade of Ca_v_2.3 VACCs by SNX-482 produced analgesic effects in the second phase of the formalin test, although it increased nociceptive behavior in the first phase [5]. These previous reports suggest that Ca_v_2.3 VACCs play differential roles in pain transmission, depending on the type of pain stimulus. In the present study, PPLpre evaluated with the cold pressor test was not associated with the genotypes of the SNPs in the *CACNA1E* gene, consistent with previous findings, and might suggest that Ca_v_2.3 VACCs are not involved in cold pain transmission under naive conditions. Furthermore, Ca_v_2.3 VACC knockout mice were reported to display enhanced morphine-induced analgesia in the tail-flick and hot-plate tests [16]. In the same report, intracerebroventricular administration of SNX-482 also enhanced morphine-induced analgesia in wildtype mice [16]. Thus, inhibition of Ca_v_2.3 VACCs may modulate the analgesic effects of opioids. In the present study, we found that fentanyl use for pain management was reduced, but the analgesic effects of fentanyl evaluated with the cold pressor test were not significantly altered in subjects with the minor G allele of the rs3845446 A/G SNP. Altogether, the rs3845446 A/G SNP or other polymorphisms in the same LD11 region may cause reduced expression levels of Ca_v_2.3 VACC mRNA and protein or dysfunctional changes (i.e., amino acid substitutions, splicing variants) and enhance the analgesic effects of opioids. However, the Ca_v_2.3 VACC inhibition-induced enhancement of opioid-induced analgesia may depend on the type of pain stimulus or pain expression (i.e., acute or chronic).

Although many SNPs have been identified in the *CACNA1E* gene, and some of these SNPs have been reported to be associated with type 2 diabetes [17], [18], we are aware of no association studies of these SNPs and pain sensitivity or opioid analgesia. The rs3845446 A/G SNP in the *CACNA1E* gene displayed a significant association with opioid analgesia, but still unknown is whether this SNP alters gene function or expression, which may be an important limitation of the present study. The rs3845446 A/G SNP represents LD11 from intron 46 to exon 47 that contains a stop codon of the *CACNA1E* gene. Interestingly, Ca_v_2.3 VACCs reportedly contain several alternative splicing variants [31], [32], [33]. The Ca_v_2.3d, e, and f isoforms contain a 45-amino-acid insertion between exons 44 and 45 (represented by the gray box in Fig. 1) in the proximal carboxy terminus, and the major Ca_v_2.3c isoform does not contain this insertion [33]. Because this splicing difference occurs between LD10 and LD11 in the *CACNA1E* gene, polymorphisms in LD10 and LD11 that contain the rs3845446 A/G SNP might affect this splicing mechanism and induce a functional reduction of neuronal transmission via Ca_v_2.3 VACCs. Further studies that focus on gene polymorphisms in LD10 and LD11 in the *CACNA1E* gene may reveal the functional mechanisms that affect pain sensitivity and the clinical efficacy of opioids.

### Conclusions

In Japanese patients who underwent sagittal split ramus osteotomy, the analgesic effects of fentanyl were related to the genotype of the *CACNA1E* gene. Subjects with the minor G allele of the rs3845446 A/G SNP required less fentanyl for adequate postoperative pain control. Although further validation is needed because the sample size of the present study was quite small, our data might provide valuable information for the appropriate individualization of fentanyl doses to achieve adequate pain control in the future.

## Supporting Information

Figure S1 **Haploview LD plot of SNPs in and around the** ***CACNA1E*** **gene.** *D*′ values are indicated in the figure.
(TIF)
