# Supplementary figures and images for: Association between Genetic Polymorphisms in Cav2.3 (R-type) Ca2+ Channels and Fentanyl Sensitivity in Patients Undergoing Painful Cosmetic Surgery
Source: PLoS One. 2013 Aug 5;8(8):e70694. doi: 10.1371/journal.pone.0070694 (PMC3734060; doi:10.1371/journal.pone.0070694)

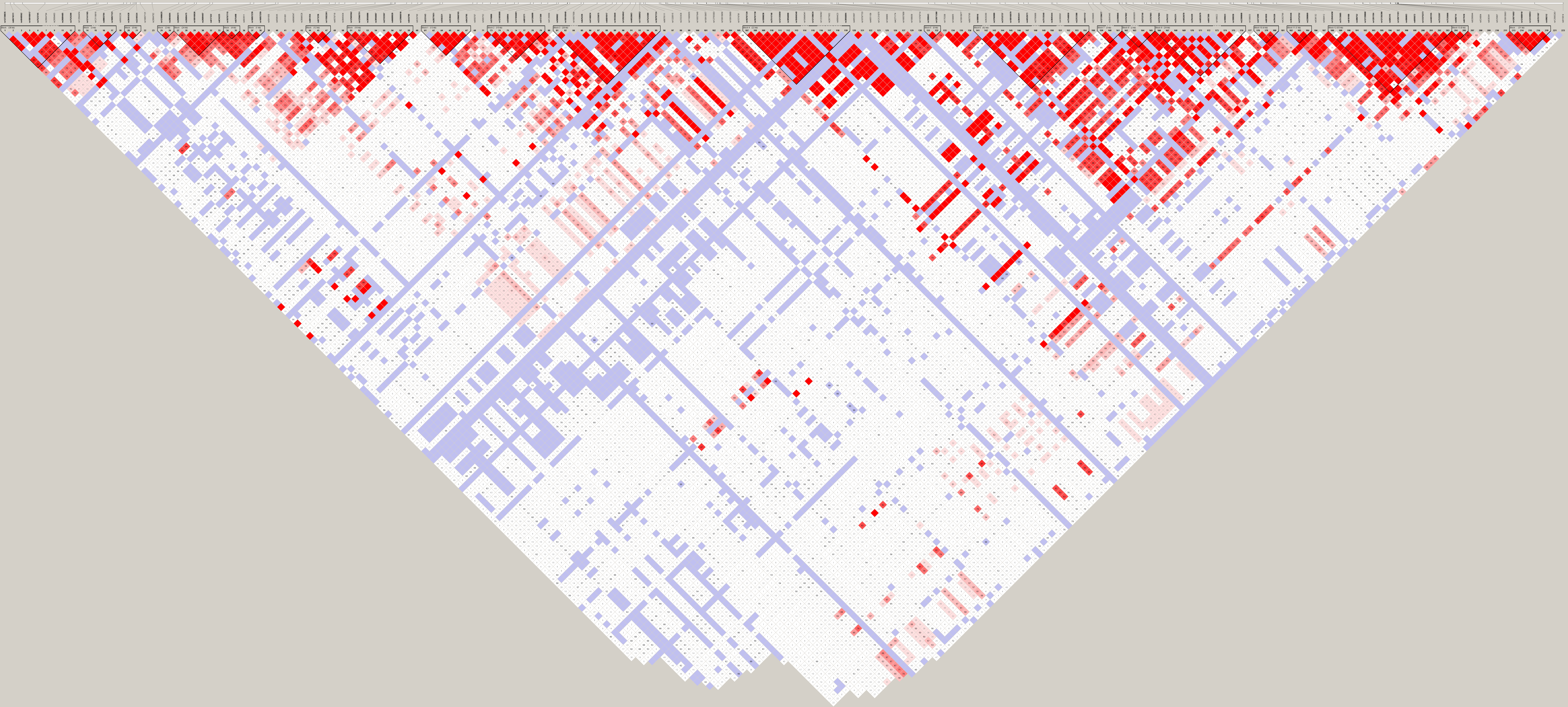

Supplement: Figure S1 — Haploview LD plot of SNPs in and around the CACNA1E gene. D′ values are indicated in the figure. (TIF) [file pone.0070694.s001.tif]
